# Character strengths and well-being across the life span: data from a representative sample of German-speaking adults living in Switzerland

**DOI:** 10.3389/fpsyg.2014.01253

**Published:** 2014-11-04

**Authors:** María L. Martínez-Martí, Willibald Ruch

**Affiliations:** ^1^Swiss National Centre of Competence in Research, LIVES – Overcoming Vulnerability: Life Course PerspectivesSwitzerland; ^2^Section on Personality and Assessment, Department of Psychology, University of ZurichZurich, Switzerland

**Keywords:** character strengths, virtues, positive psychology, VIA-IS, character strengths rating form (CSRF), well-being, representative sample

## Abstract

Character strengths are positive, morally valued traits of personality. This study aims at assessing the relationship between character strengths and subjective well-being (i.e., life satisfaction, positive and negative affect) in a representative sample of German-speaking adults living in Switzerland (*N* = 945). We further test whether this relationship is consistent at different stages in life. Results showed that hope, zest, love, social intelligence and perseverance yielded the highest positive correlations with life satisfaction. Hope, zest, humor, gratitude and love presented the highest positive correlations with positive affect. Hope, humor, zest, honesty, and open-mindedness had the highest negative correlations with negative affect. When examining the relationship between strengths and well-being across age groups, in general, hope, zest and humor consistently yielded the highest correlations with well-being. Additionally, in the 27–36 years group, strengths that promote commitment and affiliation (i.e., kindness and honesty) were among the first five positions in the ranking of the relationship between strengths and well-being. In the 37–46 years group, in addition to hope, zest and humor, strengths that promote the maintenance of areas such as family and work (i.e., love, leadership) were among the first five positions in the ranking. Finally, in the 47–57 years group, in addition to hope, zest and humor, strengths that facilitate integration and a vital involvement with the environment (i.e., gratitude, love of learning) were among the first five positions in the ranking. This study partially supports previous findings with less representative samples on the association between character strengths and well-being, and sheds light on the relative importance of some strengths over others for well-being across the life span.

## Introduction

Good character can be understood as a family of morally valued, positive traits of personality, which are relatively stable and generalizable across different situations, but which are not necessarily fixed or rooted in immutable genetic features (Peterson and Seligman, 2004). Although character has been a matter of reflection since ancient times, it has been neglected in psychology until very recently. This state of abandonment was probably due to the influence of Allport (1921), one of the most prominent figures of personality, who argued that character was not part of psychology, but it belonged to the social ethics field. However, with the emergence in the late 90s of positive psychology, the study of character regained attention in psychology and was established as a legitimate research topic.

Based on an extensive review of religious and philosophical texts, Peterson and Seligman (2004) proposed a classification of character strengths and virtues. The authors suggested the existence of six virtues, namely, wisdom, courage, humanity, justice, temperance, and transcendence. Virtues are the core characteristics of character valued by religious thinkers and philosophers. The virtue of wisdom comprises cognitive strengths that entail the acquisition and use of knowledge. The virtue of courage contains emotional strengths that involve the exercise of will to accomplish goals in the face of opposition, external or internal. The virtue of humanity includes interpersonal strengths that involve “tending and befriending” others. The virtue of justice comprises civic strengths that underlie healthy community life. The virtue of temperance contains strengths that protect against excess. And finally, the virtue of transcendence includes strengths that forge connections to the larger universe and provide meaning (Ruch et al., 2014). Moreover, each virtue comprises a number of strengths, up to a total of 24 strengths, which are the psychological ingredients that define the virtues. For example, the virtue of wisdom includes strengths such as curiosity or creativity, while the virtue of transcendence includes strengths such as hope or humor.

Empirical evidence shows that the endorsement of character strengths is significantly related to a higher degree of well-being. Usually strengths are positively correlated with life satisfaction, especially hope, zest, gratitude, curiosity and love (e.g., Park et al., 2004; Ruch et al., 2010, 2013; Buschor et al., 2013). However, some studies have shown slightly different results. For example, in a Swiss sample, Peterson et al. (2007) found that the strengths most highly correlated positively with life satisfaction were hope, zest, perseverance, and love, with social intelligence, perspective and curiosity occupying the fifth position. Also Ruch et al. (2007) found that hope, zest, love, curiosity, and perseverance were the five strengths with the highest positive correlations with life satisfaction in another Swiss sample. Lowest correlations, usually non-significant, are found for strengths such as modesty, prudence, fairness or religiousness/spirituality (e.g., Ruch et al., 2010). Other studies have shown a positive correlation with positive affect (e.g., Güsewell and Ruch, 2012; Littman-Ovadia and Lavy, 2012; Azañedo et al., 2014). Littman-Ovadia and Lavy (2012) found that the five strengths most highly correlated positively with positive affect were zest, curiosity, love of learning, hope and perspective, while the lowest correlations were observed for religiousness/spirituality (non-significant), forgiveness, modesty, prudence, and appreciation of beauty and excellence. Azañedo et al. (2014) found that zest, hope, curiosity, creativity, and perspective most highly and positively correlated with positive affect, while the lowest correlations were observed for modesty (non-significant), prudence, fairness, religiousness/spirituality, and forgiveness. For negative affect, Littman-Ovadia and Lavy (2012) found that the five strengths with the highest negative correlations were hope, curiosity, zest, love and self-regulation, while the lowest negative correlations were observed for appreciation of beauty and excellence, modesty, creativity, bravery, and prudence (all non-significant). Azañedo et al. (2014) found that the five strengths with the highest negative correlations with negative affect were hope, zest, self-regulation, persistence, gratitude, and forgiveness, while the lowest negative correlations were observed for creativity (non-significant), appreciation of beauty and excellence (non-significant), religiousness/spirituality (non-significant), modesty, and love of learning and leadership. In general, correlations were larger in size with positive affect than with negative affect in both studies. However, one limitation most studies in character strengths research share is the limited representativeness of their samples. Usually in these studies, participants are students who participate to obtain extra credits in their courses, individuals who are actively seeking how to increase their well-being, or simply convenience samples that normally result in biased samples. Therefore, we believe that studies with more representative samples are necessary in character research.

Additionally, the relationship between character strengths and well-being might be different for individuals at different stages of life, a question that still remains largely unexplored. Based on Erikson's account of stages of psychosocial development, we believe that strengths may help the individuals adapt successfully to the different stages of life, and their relative importance might be reflected in their relationship with well-being. Erikson (1982) described eight stages in psychosocial development, three of which correspond to the adult life: young adulthood, adulthood, and old age. Regarding the age range proper to these stages, according to Erikson, they are delimited by the earliest moment a developmental quality can come to relative dominance and to a meaningful crisis, and the latest at which it must yield that dominance to the next quality, although no specific ages are indicated. Young adults experience the psychosocial crisis between intimacy and isolation. Intimacy refers to the capacity to commit oneself to concrete affiliations that may call for sacrifices and compromises, while isolation is the fear of remaining separate. Intimacy must provide ways that cultivate styles of in-group living held together by idiosyncratic ways of behaving and speaking. The next stage, adulthood, is characterized by the psychosocial crisis between generativity and stagnation. According to Erikson, the spirit of adulthood is the maintenance of the world, i.e., the commitment to take care of the persons, the products, and the ideas one has learned to take care for. Finally, in old age, the psychosocial crisis is characterized by the antithesis between a sense of integrity, i.e., coherence and wholeness, vs. a sense of despair, i.e., a state of being finished, confused and helpless. Integrity seems to convey wisdom, defined by Erikson as a type of informed and detached concern with life itself in the face of death itself. For Erikson, hope is the “most basic quality of I-ness, without which life could not begin or meaningfully end.” In fact, if hope is for him the first strength emerging in infancy, faith is the mature and last possible form of hope. Also, according to Erikson, all functions specific of a life stage do not disappear in the next stage, but assume new values. In fact, old people need to keep a generativity function. However, in old age, a discontinuity of the family life contributes to the lack of the vital involvement that is necessary for staying really alive. In fact, lack of vital involvement is often the hidden reason that brings old people to psychotherapy (Erikson, 1982). Considering Erikson's theory, strengths that help fulfill the specific functions of each stage of life, should have a larger relationship with well-being in that stage in comparison with other strengths.

Nonetheless, empirical evidence on this topic is almost non-existent. As far as we know, only one study has explored the relationship between character strengths and well-being (specifically, life satisfaction) across different age groups. Isaacowitz et al. (2003) suggested that strengths that help individuals explore the world and protect them from difficulties should be more strongly related to well-being for young adults. Strengths related to building a career and a family should be more strongly associated with well-being for middle-aged individuals. Finally, strengths that contribute to keep social relationships should be more strongly related to well-being in older individuals. Also, since older individuals regulate their emotions better than younger individuals, Isaacowitz et al. (2003) also suggested that strengths related to temperance and control should be more important for the well-being of older individuals. Additionally, since older adults do not have to invest so much time and effort in raising a family and building a career, they might have more opportunities to apply their strengths and thus strengths would be more strongly related to well-being. Using a narrower classification of strengths than the classification proposed by Peterson and Seligman (2004), Isaacowitz et al. (2003) observed that for young adults, only hope significantly predicted life satisfaction. For middle-aged individuals, only the capacity for loving relationships predicted life satisfaction. For community older adults, only the strengths of hope, citizenship, and loving relationships predicted life satisfaction. Although we embrace the empirical evidence that this study provides, unfortunately Isaacowitz et al. (2003) used a classification of strengths different from Peterson and Seligman's (2004) classification, and the samples used were not representative of the population.

In order to fulfill the need of studies that test the relationship between character strengths and well-being in more representative samples, and, considering the virtual non-existence of studies assessing this relationship across the life span, the aim of this study is twofold. First, we examined the relationship between character strengths and subjective well-being (i.e., life satisfaction, positive affect, and negative affect) in a representative sample of German-speaking adults living in Switzerland. Using a more representative sample will provide a more accurate account of the relationship between character strengths and well-being. In general, we expect strengths to correlate positively with life satisfaction and positive affect, and negatively with negative affect. Moreover, based on previous studies (e.g., Park et al., 2004; Peterson et al., 2007; Ruch et al., 2010), we predict that, in general, strengths such as hope, zest, love, curiosity, gratitude, perseverance, social intelligence and perspective might yield the highest positive correlations with life satisfaction, while strengths such as modesty, prudence, fairness or spirituality/religiousness might show the lowest positive correlations. Also based on previous evidence (i.e., Littman-Ovadia and Lavy, 2012; Azañedo et al., 2014), positive affect might be more highly and positively correlated with zest, curiosity, hope, love of learning, creativity, and perspective, while lowest positive correlations are expected with modesty, prudence, fairness, spirituality/religiousness, forgiveness, and appreciation of beauty and excellence. On the other hand, negative affect might yield the highest negative correlations with hope, zest, curiosity, self-regulation, love, persistence, gratitude, and forgiveness, while the lowest negative correlations are expected with creativity, appreciation of beauty and excellence, spirituality/religiousness, modesty, love of learning, leadership, bravery, and prudence. Similarly, according to prior data (i.e., Littman-Ovadia and Lavy, 2012; Azañedo et al., 2014), it is possible that the relationship with the positive indicators of well-being (i.e., life satisfaction and positive affect) is, overall, larger in size than with the negative indicator (i.e., negative affect).

The second goal of this study is to examine the relationship between strengths and subjective well-being (i.e., life satisfaction, positive affect and negative affect) across the life span. Differences in the relationship between strengths and well-being across the life span can have important implications not only for character strengths theory, but also for strengths-based interventions. Depending on the age of the clients, these interventions might be helpful in the attempt to focus especially on the strengths most highly associated with well-being in the corresponding life stage, in order to improve the person-fit and increase their efficacy. Building upon Isaacowitz et al.'s (2003) study and Erikson's theory of the stages of psychosocial development, we believe that hope might be especially relevant at all stages, although it may take another form in old age, i.e., faith. Although strengths that help forge social connections should be important at all stages, we believe that especially for young adults, strengths that promote the commitment and affiliation with others, such as honesty, kindness, social intelligence, teamwork, gratitude, humor or love, might yield larger correlations with well-being in comparison to other strengths. For adults, we think that strengths that support the maintenance of the world (i.e., take care of people, products, and ideas), such as perseverance, love or leadership, might present larger correlations with well-being in comparison to other strengths. And finally, for old adults, who have lived most of their life and have a greater awareness of finitude, strengths that enable individuals to integrate the past into the present, such as gratitude and forgiveness, and allow them to transcend themselves in order to feel part of a broader reality, strengths such as spirituality/religiousness, might be particularly advantageous. Also, strengths that enable one to keep an active lifestyle, such as zest, love of learning, or curiosity, and a positive outlook of life, such as gratitude, hope, or humor, might be particularly beneficial for old adults. Additionally, according to Isaacowitz et al. (2003), since old adults might be more free than young adults from the need to build resources for the future, and fulfill professional and family responsibilities, it is possible that old adults can actually apply more of their strengths in their lives, and thus, strengths in general might yield higher correlations with well-being in the old adults. According to Erikson's theory, the functions typical of each life stage do not disappear in the next stage but change their values. Therefore, in old age there might be more functions to fulfill and more character strengths may be helpful in fulfilling these functions. Thus, strengths might yield a higher positive correlation with well-being in old adults than in younger individuals.

## Materials and methods

### Participants

A representative sample of 945 German-speaking adults of working age (459 men, 486 women) living in Switzerland participated in this study. The age of participants ranged from 27 to 57 years (*M* = 43.60, *SD* = 8.62). Most participants were Swiss (*n* = 792), 152 had other nationalities different from Swiss, and one person did not report the nationality. Most participants were married or in a relationship (*n* = 536), 261 were single, 87 were divorced, 22 were separated, nine were widowed, and 30 reported to be in an “other” situation. Regarding the educational level, 309 participants had completed tertiary education (e.g., university), 487 had finished secondary education (e.g., vocational training or high school), 33 had finished primary school, one had not finished primary school, 52 reported an “other” education level, and 63 were missing values. Concerning religion, 296 belonged to the Protestant church/Evangelical reformed, 18 to other Evangelical communities and Free churches, 297 to the Roman Catholic church, eight to the Christian Catholic church, 17 to the Orthodox Christian church, eight to other Christian communities, 18 to Islamic communities, 12 to other churches and religious communities, 208 did not have any religious affiliation, and 63 did not list their religious community/preferences. Three age subgroups were created. The first group (*n* = 241) comprised participants with ages ranging from 27 to 36, including participants with 27 and 36 years old. The second group (*n* = 301) consisted of participants with ages ranging from 37 to 46, including participants with 37 and 46 years old. Finally, the third group (*n* = 403) comprised participants with ages ranging from 47 to 57, including participants with 47 and 57 years old.

### Instruments

The *Character Strengths Rating Form* (*CSRF*; Ruch et al., 2014) is a 24-item rating form of character strengths, based on the classification proposed by Peterson and Seligman (2004). It uses a 9-point Likert scale (1, totally inaccurate; 2, inaccurate; 3, largely inaccurate; 4, partially inaccurate; 5, neither one nor the other; 6, partially accurate; 7, largely accurate; 8, accurate; 9, completely accurate). Each item is a description of a strength and measures the endorsement of that specific strength. For example, the item measuring creativity is: “Creativity (originality, ingenuity): Creative people have a highly developed thinking about novel and productive ways to solve problems and often have creative and original ideas. They do not content themselves with conventional solutions if there are better solutions.” The German version was used. The CSRF has shown good convergence with the Values in Action Inventory of Strengths (VIA-IS, Peterson and Seligman, 2004), in terms of descriptive statistics, relationships with socio-demographic variables and life satisfaction, and factor structure (Ruch et al., 2014). The development of the CSRF was motivated by the need to include a short measure of character strengths in a large-scale longitudinal study, i.e., the NCCR-LIVES project (*Swiss National Centre of Competence in Research LIVES—Overcoming vulnerability: Life course perspectives*), which studies the impact of vulnerabilities and strengths on life over time.

The *Satisfaction with Life Scale* (*SWLS*; Diener et al., 1985) is a 5-item questionnaire for the subjective assessment of global life satisfaction (e.g., “I am satisfied with my life”), utilizing a 7-point answer format (1, strongly disagree; 2, disagree; 3, slightly disagree; 4, neither disagree nor agree; 5, slightly agree; 6, agree; 7, strongly agree). We used the German version used by Ruch et al. (2010), which was developed in a standardized translation-back-translation-procedure, and has shown good psychometric properties. Cronbach alpha in the present study was 0.91.

The *Affect Scale* of the *Midlife Development Inventory* (*MIDI-Affect*; Mroczek and Kolarz, 1998) is a 12-item questionnaire for the assessment of positive affect (6 items, e.g., “During the last month, how much of the time did you feel cheerful?”) and negative affect (6 items, e.g., “During the last month, how much of the time did you feel hopeless?”). A 5-point answer format is used (1, none of the time; 2, rarely; 3, from time to time; 4, most of the time; 5, all of the time). Positive and negative affect scales have shown internal consistencies of α = 0.91 and α = 0.87 respectively (Mroczek and Kolarz, 1998). A German version of the scale was used, which was translated following a standardized translation-back-translation-procedure, which is described below. Cronbach alphas in the present study were 0.85 for positive affect and 0.82 for negative affect.

### Procedure

We present data from a project focused on the impact of individual characteristics, resources and cultural background on professional trajectories; this project is part of the NCCR-LIVES (*Swiss National Centre of Competence in Research LIVES—Overcoming vulnerability: Life course perspectives*). In this ongoing longitudinal project, data are collected over seven consecutive years from a representative sample of individuals living in Switzerland (Maggiori et al., in press). We present some data from the second wave of data collection, carried out in 2013. The recruitment was done on the base of a representative sample of subjects with ages ranging between 26 and 56 years drawn from the Swiss national register of inhabitants and conducted by the Swiss Federal Statistics Office. An institute specialized in research surveys conducted the data collection. First, participants received a letter with the study description. Then, the first part of the survey (sociodemographic data and employment-related information) was performed either by phone or online (participants could choose the method), and the second part (remaining questionnaires) was conducted either using a paper and pencil method, or online. Participants answered the survey at home or at any place they wished. The time of survey completion was approximately 40 to 55 min, but participants did not have any limit in this regard. When necessary, the instruments used in the survey were translated from the original language into German. Two independent translations in German were done by bilingual psychologists, and combined into one. This translation was then checked and back-translated into English. The comparison of the two versions was done by the original author/translator. Finally, the research survey institute in charge of the data collection checked the final versions. This study fulfills the ethical standards for research of the Swiss Society for Psychology. Participants' anonymity was preserved. The institute specialized in research surveys which conducted the data collection kept the personal information and researchers received only a dataset in which participants were assigned numerical codes, and no personal information. Also, once the research project is finished, all personal information will be destroyed (expected to be completed in 2018). Participants are free to withdraw from the study at any time. Participants received a gift for a value of 20 Swiss francs for their participation.

## Results

### Descriptive statistics

Table 1 shows the means and standard deviations of the 24 strengths and the well-being indicators in the total sample, and across age categories.

**Table 1:** **Means and standard deviations of strengths and well-being indicators in the total sample, and across age groups**.

	**Total**		**Age 27–36**		**Age 37–46**		**Age 47–57**	
	***M***	***SD***	***M***	***SD***	***M***	***SD***	***M***	***SD***
**STRENGTHS**								
Creativity	6.39	1.86	6.32	1.86	6.29	1.89	6.51	1.84
Curiosity	6.90	1.61	6.89	1.53	6.83	1.64	6.97	1.64
Open-mindedness	6.79	1.53	6.76	1.47	6.70	1.62	6.88	1.49
Love Learning	6.69	1.61	6.66	1.60	6.70	1.54	6.69	1.67
Perspective	6.54	1.56	6.59	1.49	6.53	1.53	6.52	1.62
Bravery	6.27	1.69	6.26	1.70	6.22	1.70	6.32	1.68
Perseverance	6.79	1.56	6.71	1.54	6.76	1.65	6.87	1.49
Honesty	7.41	1.40	7.40	1.44	7.41	1.44	7.43	1.34
Zest	6.33	1.61	6.37	1.60	6.32	1.63	6.31	1.61
Love	6.96	1.54	7.01	1.53	6.74	1.60	7.09	1.49
Kindness	7.21	1.35	7.40	1.31	7.14	1.40	7.14	1.34
Social intelligence	7.08	1.42	7.19	1.41	6.98	1.48	7.08	1.38
Teamwork	6.81	1.55	6.90	1.53	6.84	1.51	6.72	1.60
Fairness	7.20	1.37	7.15	1.39	7.21	1.38	7.22	1.35
Leadership	6.49	1.72	6.48	1.64	6.46	1.82	6.53	1.68
Forgiveness	6.72	1.54	6.69	1.47	6.68	1.62	6.77	1.53
Modesty	6.24	1.76	6.19	1.73	6.30	1.69	6.22	1.84
Prudence	6.25	1.68	6.13	1.73	6.37	1.65	6.23	1.68
Self-regulation	5.85	1.77	5.81	1.86	5.90	1.73	5.84	1.74
Appreciation Beauty	6.56	1.61	6.46	1.60	6.49	1.60	6.67	1.61
Gratitude	6.78	1.46	6.85	1.47	6.80	1.45	6.71	1.47
Hope	6.86	1.50	6.97	1.41	6.82	1.53	6.83	1.52
Humor	6.86	1.60	7.09	1.44	6.87	1.57	6.71	1.69
Spirituality/Religiousness	5.03	2.41	4.82	2.36	4.90	2.43	5.25	2.40
**WELL-BEING**								
Life satisfaction	5.19	1.17	5.19	1.17	5.18	1.22	5.20	1.12
Positive affect	3.63	0.56	3.61	0.55	3.62	0.56	3.64	0.58
Negative affect	1.92	0.59	1.98	0.59	1.94	0.61	1.87	0.58

We carried out a series of one-way analyses of variance to explore differences in character strengths and well-being between different age groups. Age groups significantly differed in love *F*_(2, 942)_ = 4.73, *p* = 0.009, kindness, *F*_(2, 942)_ = 3.15, *p* = 0.043, humor, *F*_(2, 942)_ = 4.31, *p* = 0.014, and spirituality/religiousness, *F*_(2, 942)_ = 3.12, *p* = 0.044. Post hoc comparisons with Bonferroni correction showed that the 27–36 years group scored significantly higher in kindness (*p* = 0.064, *d* = 0.20) and humor (*p* = 0.010, *d* = 0.15) than the 47–57 years group. On the other hand, the 47–57 years group scored significantly higher than the 27–36 years group in love (*p* = 0.008, *d* = 0.05) and religiousness (*p* = 0.078, *d* = 0.18). Regarding differences in well-being, age groups did not differ significantly.

### Correlations between strengths and well-being

The correlations between strengths and well-being in the total sample are presented in Table 2. This table also shows the rank order of these correlations for each indicator of well-being, and the mean absolute values of these correlations across strengths and across indicators.

**Table 2:** **Correlations between strengths and different indicators of well-being, rank order, and mean absolute values of these correlations across strengths (columns) and well-being indicators (rows)**.

**Strengths**	***SWL***	***R***	***PA***	***R***	***NA***	***R***	***M***	***R***
Creativity	0.13^*^	14	0.15^*^	13	−0.09^*^	16	0.12	14
Curiosity	0.15^*^	8	0.17^*^	10	−0.11^*^	8	0.14	8
Open-mindedness	0.11^*^	16	0.11^*^	20	−0.13^*^	5	0.12	16
Love Learning	0.17^*^	6	0.15^*^	14	−0.11^*^	10	0.14	11
Perspective	0.15^*^	9	0.14^*^	18	−0.07	19	0.12	15
Bravery	0.10^*^	18	0.14^*^	16	−0.10^*^	13	0.11	18
Perseverance	0.19^*^	5	0.20^*^	6	−0.12^*^	6	0.17	5
Honesty	0.14^*^	12	0.14^*^	17	−0.14^*^	4	0.14	10
Zest	0.26^*^	2	0.32^*^	2	−0.16^*^	3	0.25	2
Love	0.23^*^	3	0.20^*^	5	−0.10^*^	12	0.18	4
Kindness	0.15^*^	10	0.18^*^	8	−0.07	17	0.13	12
Social intelligence	0.20^*^	4	0.17^*^	9	−0.12^*^	7	0.16	6
Teamwork	0.12^*^	15	0.17^*^	11	−0.10^*^	11	0.13	13
Fairness	0.05	21	0.10^*^	19	−0.06	20	0.07	20
Leadership	0.14^*^	11	0.18^*^	7	−0.11^**^	9	0.14	9
Forgiveness	0.08	19	0.16^*^	12	−0.07	18	0.10	19
Modesty	−0.03	24	0.03	24	0.01	23	0.02	24
Prudence	0.06	20	0.08	22	−0.04	21	0.06	21
Self-regulation	0.11^*^	17	0.15^*^	15	−0.09^*^	15	0.11	17
Appreciation Beauty	0.05	22	0.10^*^	21	−0.01	22	0.05	22
Gratitude	0.16^*^	7	0.22^*^	4	−0.09^*^	14	0.16	7
Hope	0.31^*^	1	0.35^*^	1	−0.25^*^	1	0.30	1
Humor	0.14^*^	13	0.27^*^	3	−0.17^*^	2	0.19	3
Spirituality/Religiousness	0.03	23	0.03	23	0.04	24	0.03	23
M/R	0.14	2	0.16	1	0.10	4	0.13	

*N = 945. SWL, satisfaction with life; PA, positive affect; NA, negative affect; M, mean absolute value; R, rank order*.

* *p < 0.01*.

** *p < 0.001*.

Additionally, we examined whether these correlations varied across age groups (see Table 3 for participants with ages between 27 and 36 years, Table 4 for participants with ages between 37 and 46 years, and Table 5 for participants with ages between 47 and 57 years).

**Table 3:** **Correlations between strengths and well-being in the 27–36 years group (*n* = 241)**.

**Strengths**	***SWL***	***R***	***PA***	***R***	***NA***	***R***	***M***	***R***
Creativity	0.10	17	0.04	18	−0.00	18	0.05	20
Curiosity	0.14	10	0.15	8	−0.01	17	0.10	13
Open-mindedness	0.12	13	0.09	14	−0.05	12	0.09	15
Love Learning	0.13	12	0.08	15	−0.06	9	0.09	14
Perspective	0.12	14	0.05	17	0.02	20	0.06	18
Bravery	0.01	22	0.03	20	−0.00	19	0.01	24
Perseverance	0.16	9	0.13	10	−0.09	7	0.12	10
Honesty	0.16	8	0.16	7	−0.16	2	0.16	5
Zest	0.28^*^	2	0.24^*^	3	−0.09	6	0.21	2
Love	0.23^*^	5	0.13	9	−0.02	15	0.13	9
Kindness	0.25^*^	3	0.19^*^	4	−0.06	11	0.17	4
Social intelligence	0.25^*^	4	0.11	11	−0.10	5	0.15	6
Teamwork	0.10	16	0.18^*^	5	−0.13	4	0.14	7
Fairness	0.06	20	0.09	13	−0.06	10	0.07	16
Leadership	0.14	11	0.10	12	−0.07	8	0.10	12
Forgiveness	0.02	21	−0.01	22	0.03	22	0.02	23
Modesty	0.00	23	−0.09	23	0.02	21	0.04	22
Prudence	0.11	15	0.04	19	−0.05	13	0.07	17
Self-regulation	0.08	18	0.05	16	−0.02	16	0.05	19
Appreciation Beauty	0.07	19	−0.00	21	0.04	23	0.04	21
Gratitude	0.16	6	0.16	6	−0.03	14	0.12	11
Hope	0.31^*^	1	0.26^*^	1	−0.31^*^	1	0.29	1
Humor	0.16	7	0.25^*^	2	−0.14	3	0.18	3
Spirituality/Religiousness	−0.06	24	−0.20^*^	24	0.15	24	0.13	8
M/R	0.13	1	0.12	2	0.07	4	0.11	

*SWL, satisfaction with life; PA, positive affect; NA, negative affect; M, mean absolute value; R, rank order*.

* *p < 0.01*.

**Table 4:** **Correlations between strengths and well-being in the 37–46 years group (*n* = 301)**.

**Strengths**	***SWL***	***R***	***PA***	***R***	***NA***	***R***	***M***	***R***
Creativity	0.14	9	0.11	17	−0.08	17	0.12	13
Curiosity	0.20^*^	5	0.16^*^	11	−0.17^*^	7	0.18	7
Open-mindedness	0.09	13	0.07	22	−0.21^*^	4	0.12	12
Love Learning	0.16^*^	7	0.09	20	−0.12	13	0.12	11
Perspective	0.14	11	0.14	13	−0.10	15	0.13	10
Bravery	0.07	17	0.15	12	−0.10	14	0.11	17
Perseverance	0.22^*^	3	0.24^*^	4	−0.14	10	0.20	6
Honesty	0.08	14	0.10	19	−0.14	11	0.11	18
Zest	0.22^*^	4	0.32^*^	2	−0.17^*^	5	0.24	2
Love	0.27^*^	2	0.24^*^	5	−0.15^*^	8	0.22	3
Kindness	0.08	16	0.13	14	−0.10	16	0.10	19
Social intelligence	0.18^*^	6	0.16	10	−0.15^*^	9	0.16	8
Teamwork	0.07	18	0.10	18	−0.09	19	0.09	20
Fairness	0.08	15	0.11	16	−0.13	12	0.11	16
Leadership	0.16^*^	8	0.23^*^	6	−0.21^*^	3	0.20	5
Forgiveness	0.05	21	0.21^*^	8	−0.09	18	0.12	14
Modesty	−0.04	24	0.08	21	−0.03	22	0.05	22
Prudence	0.05	20	0.13	15	−0.09	20	0.09	21
Self-regulation	0.07	19	0.21^*^	7	−0.17^*^	6	0.15	9
Appreciation Beauty	−0.03	23	0.03	24	0.03	24	0.03	23
Gratitude	0.09	12	0.16^*^	9	−0.08	21	0.11	15
Hope	0.30^*^	1	0.39^*^	1	−0.30^*^	1	0.33	1
Humor	0.14	10	0.28^*^	3	−0.23^*^	2	0.22	4
Spirituality/Religiousness	−0.00	22	0.07	23	−0.02	23	0.03	24
M/R	0.12	4	0.16	1	0.13	3	0.14	

*SWL, satisfaction with life; PA, positive affect; NA, negative affect; M, mean absolute value; R, rank order*.

* *p < 0.01*.

**Table 5:** **Correlations between strengths and well-being in the 47–57 years group (*n* = 403)**.

**Strengths**	***SWL***	***R***	***PA***	***R***	***NA***	***R***	***M***	***R***
Creativity	0.15^*^	13	0.24^*^	5	−0.12	8	0.17	9
Curiosity	0.12	19	0.18^*^	17	−0.12	10	0.14	16
Open-mindedness	0.13	17	0.14^*^	20	−0.11	11	0.13	18
Love Learning	0.20^*^	5	0.23^*^	6	−0.12	7	0.18	5
Perspective	0.18^*^	7	0.19^*^	14	−0.11	12	0.16	12
Bravery	0.17^*^	10	0.21^*^	9	−0.15^*^	4	0.17	6
Perseverance	0.20^*^	6	0.20^*^	13	−0.12	9	0.17	7
Honesty	0.18^*^	8	0.17^*^	18	−0.14^*^	6	0.16	11
Zest	0.29^*^	2	0.36^*^	2	−0.18^*^	2	0.28	2
Love	0.21^*^	4	0.21^*^	10	−0.10	16	0.17	8
Kindness	0.15^*^	14	0.21^*^	11	−0.07	18	0.14	15
Social intelligence	0.17^*^	9	0.21^*^	8	−0.11	13	0.17	10
Teamwork	0.17^*^	11	0.21^*^	12	−0.10	15	0.16	13
Fairness	0.01	23	0.11^*^	22	−0.00	22	0.04	22
Leadership	0.13	16	0.18^*^	16	−0.04	20	0.12	20
Forgiveness	0.14^*^	15	0.22^*^	7	−0.10	14	0.15	14
Modesty	−0.04	24	0.05	24	0.02	23	0.04	23
Prudence	0.03	22	0.06	23	−0.00	21	0.03	24
Self-regulation	0.16^*^	12	0.17^*^	19	−0.07	17	0.13	17
Appreciation Beauty	0.10	20	0.19^*^	15	−0.07	19	0.12	19
Gratitude	0.23^*^	3	0.29^*^	3	−0.15^*^	5	0.22	3
Hope	0.32^*^	1	0.36^*^	1	−0.19^*^	1	0.29	1
Humor	0.13	18	0.28^*^	4	−0.15^*^	3	0.18	4
Spirituality/Religiousness	0.11	21	0.13^*^	21	0.03	24	0.09	21
M/R	0.15	2	0.20	1	0.10	5	0.15	

*SWL, satisfaction with life; PA, positive affect; NA, negative affect; M, mean absolute value; R, rank order*.

* *p < 0.01*.

To compare the rank order of the relationships between character strengths and either life satisfaction, positive affect or negative affect across age groups, we calculated a series of Spearman correlations. In life satisfaction, when comparing the rank order of the 27–36 years group and the 37–46 years, the Spearman correlation was 0.77. When comparing the 27–36 years group and the 47–57 years group, the Spearman correlation was 0.64. And finally, when comparing the 37–46 years group and the 47–57 years group, the Spearman correlation was 0.66. In positive affect, when comparing the 27–36 years group and the 37–46 years group, the Spearman correlation was 0.56. When comparing the 27–36 years group and the 47–57 years, the Spearman correlation was 0.50. Finally, when comparing the 37–46 years group and the 47–57 years group, the Spearman correlation was 0.56. In negative affect, when comparing the 27–36 years group and the 37–46 years group, the Spearman correlation was 0.62. When comparing the 27–36 years group and the 47–57 years group, the Spearman correlation was 0.46. And finally, when comparing the 37–46 years group and the 47–57 years group, the Spearman correlation was 0.47.

Additionally, in order to test whether the size of the correlations between strengths and well-being were statistically different among the three age groups, we conducted a series of *Z* test. In order to control for the number of comparisons performed, here we only report the comparisons that were significantly different at *p* < 0.01. The remaining *Z* tests are available in the Table 6, in the supplementary material of this article. The correlation between positive affect and creativity was significantly larger in the 47–57 years group than in the 27–36 years group (*Z* = 2.49, *p* = 0.006). Also, the correlation between positive affect and forgiveness was significantly larger in the 37–46 years group (*Z* = 2.50, *p* = 0.006) and in the 47–57 years group (*Z* = 2.87, *p* = 0.002) than in the 27–36 years group. Likewise, the correlation between positive affect and religiousness was significantly different in the 27–36 years group than the correlation in the 37–46 years group (*Z* = 3.06, *p* = 0.001) and in the 47–57 years group (*Z* = 4.05, *p* < 0.001). The remaining comparisons were not significantly different at *p* < 0.01.

**Table 6:** ***Z*-tests and associated p values for comparing the correlations between strengths and well-being indicators across age groups**.

	**Life satisfaction**						**Positive affect**						**Negative affect**					
	**1 vs. 2**		**1 vs. 3**		**2 vs. 3**		**1 vs. 2**		**1 vs. 3**		**2 vs. 3**		**1 vs. 2**		**1 vs. 3**		**2 vs. 3**	
	***Z***	***P***	***Z***	***p***	***Z***	***p***	***Z***	***p***	***Z***	***p***	***Z***	***p***	***Z***	***p***	***Z***	***p***	***Z***	***p***
1	−0.54	0.29	−0.65	0.26	−0.08	0.47	−0.75	0.23	−2.49	0.01	−1.81	0.04	1.10	0.14	1.46	0.07	0.32	0.37
2	−0.68	0.25	0.29	0.39	1.07	0.14	−0.07	0.47	−0.28	0.39	−0.22	0.41	1.84	0.03	1.33	0.09	−0.67	0.25
3	0.36	0.36	−0.10	0.46	−0.52	0.30	0.20	0.42	−0.72	0.24	−0.99	0.16	1.83	0.03	0.74	0.23	−1.29	0.10
4	−0.41	0.34	−0.90	0.18	−0.50	0.31	−0.16	0.44	−1.92	0.03	−1.87	0.03	0.68	0.25	0.76	0.22	0.04	0.48
5	−0.25	0.40	−0.80	0.21	−0.58	0.28	−1.05	0.15	−1.75	0.04	−0.69	0.25	1.43	0.08	1.58	0.06	0.07	0.47
6	−0.75	0.23	−1.95	0.03	−1.23	0.11	−1.40	0.08	−2.27	0.01	−0.84	0.20	1.15	0.13	1.77	0.04	0.58	0.28
7	−0.74	0.23	−0.52	0.30	0.29	0.39	−1.35	0.09	−0.98	0.16	0.48	0.32	0.61	0.27	0.38	0.35	−0.28	0.39
8	0.86	0.19	−0.21	0.42	−1.21	0.11	0.66	0.25	−0.14	0.44	−0.89	0.19	−0.22	0.41	−0.22	0.41	0.01	0.50
9	0.80	0.21	−0.13	0.45	−1.05	0.15	−0.95	0.17	−1.51	0.07	−0.53	0.30	0.93	0.18	1.11	0.13	0.13	0.45
10	−0.44	0.33	0.28	0.39	0.80	0.21	−1.24	0.11	−0.96	0.17	0.39	0.35	1.52	0.06	0.97	0.17	−0.69	0.25
11	2.12	0.02	1.38	0.08	−0.94	0.17	0.72	0.24	−0.19	0.42	−1.02	0.15	0.49	0.31	0.18	0.43	−0.36	0.36
12	0.82	0.21	0.99	0.16	0.12	0.45	−0.62	0.27	−1.38	0.08	−0.77	0.22	0.63	0.26	0.11	0.46	−0.60	0.27
13	0.37	0.36	−0.77	0.22	−1.25	0.11	0.94	0.17	−0.27	0.39	−1.35	0.09	−0.49	0.31	−0.35	0.36	0.18	0.43
14	−0.22	0.41	0.56	0.29	0.85	0.20	−0.29	0.39	−0.22	0.41	0.09	0.46	0.86	0.19	−0.68	0.25	−1.71	0.04
15	−0.21	0.42	0.19	0.42	0.44	0.33	−1.63	0.05	−1.10	0.14	0.68	0.25	1.63	0.05	−0.33	0.37	−2.21	0.01
16	−0.28	0.39	−1.40	0.08	−1.19	0.12	−2.50	0.01	−2.87	0.00	−0.23	0.41	1.40	0.08	1.62	0.05	0.15	0.44
17	0.53	0.30	0.46	0.32	−0.10	0.46	−1.91	0.03	−1.69	0.05	0.37	0.36	0.56	0.29	0.07	0.47	−0.56	0.29
18	0.73	0.23	1.04	0.15	0.29	0.39	−1.02	0.15	−0.33	0.37	0.80	0.21	0.44	0.33	−0.55	0.29	−1.09	0.14
19	0.10	0.46	−1.05	0.15	−1.25	0.11	−1.85	0.03	−1.40	0.08	0.61	0.27	1.73	0.04	0.66	0.25	−1.26	0.10
20	0.82	0.21	−0.44	0.33	−1.82	0.03	−0.40	0.34	−2.36	0.01	−2.07	0.02	0.10	0.46	1.32	0.09	1.30	0.10
21	0.82	0.21	−0.81	0.21	−1.80	0.04	−0.01	0.50	−1.60	0.05	−1.70	0.04	0.62	0.27	1.49	0.07	0.89	0.19
22	0.22	0.41	−0.15	0.44	−0.40	0.34	−1.76	0.04	−1.40	0.08	0.50	0.31	−0.14	0.44	−1.47	0.07	−1.42	0.08
23	0.24	0.41	0.44	0.33	0.20	0.42	−0.40	0.34	−0.35	0.36	0.07	0.47	1.01	0.16	0.12	0.45	−1.02	0.15
24	−0.66	0.25	−2.00	0.02	−1.39	0.08	−3.06	0.00	−4.05	0.00	−0.86	0.19	1.99	0.02	1.46	0.07	−0.71	0.24

*1 vs. 2, difference between the 27–36 years group and the 37–46 years group; 1 vs. 3, difference between the 27–36 years group and the 47–57 years group; 2 vs. 3, difference between the 37–46 years group and the 37–46 years group; 1, creativity; 2, curiosity; 3, open-mindedness; 4, love of learning; 5, perspective; 6, bravery; 7, perseverance; 8, honesty; 9, zest; 10, love; 11, kindness; 12, social intelligence; 13, teamwork; 14, fairness; 15, leadership; 16, forgiveness; 17, modesty; 18, prudence; 19, self-regulation; 20, appreciation of beauty and excellence; 21, gratitude; 22, hope; 23, humor. 24, spirituality/religiousness*.

## Discussion

This study offers novel evidence of the relationship between character strengths and subjective well-being in a representative sample of German-speaking adults living in Switzerland, as well as of this relationship across three different age groups.

The first goal of the study was to test whether the relationship between character strengths and subjective well-being observed in previous studies held in a more representative sample. The first five strengths most highly correlated, positively and significantly, with life satisfaction in the sample used in this study were hope, zest, love, social intelligence, and perseverance; this is highly consistent with previous findings in studies using Swiss samples and with our assumptions (e.g., Peterson et al., 2007; Ruch et al., 2007). This result is also relatively consistent with previous findings conducted with samples from other countries, where hope, zest, gratitude, curiosity and love usually yield the highest positive, significant correlations with life satisfaction (e.g., Park et al., 2004). In our sample, gratitude and curiosity held the 7th and 8th positions, respectively, i.e., relatively high positions in this ranking. Moreover, curiosity was among the five first strengths in this ranking in the 37–46 years group, and gratitude in the 47–57 years group. Although the profile observed in our study could seem as representative of the Swiss profile, as it converges very well with previous studies conducted with Swiss samples, other studies with Swiss samples have found results similar to the ones reported in most of the studies conducted on the relationship between character strengths and life satisfaction. For example, Buschor et al. (2013) found that hope, zest, love, curiosity, and gratitude were the five strengths with the highest positive correlations with life satisfaction. Nonetheless, it is worth noting that, in this same study, peer-ratings of the relationship between character strengths and life satisfaction showed that hope, zest, curiosity, perseverance, and humor were the five strengths with the highest positive correlations with life satisfaction. Thus, perseverance appears again as one the strengths most highly correlated positively with life satisfaction in a Swiss sample. When considering the lowest correlations with life satisfaction, modesty, religiousness, appreciation of beauty and excellence, fairness, and prudence showed the lowest correlations, all non-significant, which is highly consistent with previous findings and with our expectations (e.g., Park et al., 2004; Ruch et al., 2007, 2010).

In relation to positive affect, hope, zest, humor, gratitude and love were the strengths most highly correlated, positively and significantly, with positive affect, what is only partially consistent with previous research and with our assumptions (e.g., Littman-Ovadia and Lavy, 2012; Azañedo et al., 2014). Azañedo et al. (2014) and Littman-Ovadia and Lavy (2012), found that, besides hope and zest, strengths related to the use and acquisition of knowledge, such as curiosity, creativity, perspective or love of learning, yielded the highest positive correlations with positive affect. The lowest correlations with positive affect were for modesty (non-significant), religiousness (non-significant), appreciation of beauty and excellence (non-significant), prudence (significant) and open-mindedness (significant), which is highly consistent with previous findings and with our hypotheses (Littman-Ovadia and Lavy, 2012). On the other hand, in our study, aside from hope and zest, which are common to the other studies just discussed, humor, gratitude and love yielded the highest positive and significant correlations with positive affect, which make sense conceptually, given their more emotional nature.

Regarding negative affect, the five strengths most highly correlated negatively with negative affect were hope, humor, zest, honesty, and open-mindedness (all significant), which only partially agreed with the findings reported in previous studies and our assumptions (e.g., Littman-Ovadia and Lavy, 2012; Azañedo et al., 2014). Littman-Ovadia and Lavy (2012) found that negative affect yielded the highest negative correlations with hope, curiosity, zest, love, and self-regulation, while Azañedo et al. (2014) found that hope, zest, self-regulation, persistence, gratitude, and forgiveness showed the highest negative correlations. However, our results make sense and can be interpreted. Humor can be used as a coping strategy to reduce negative affect (Martin and Lefcourt, 1983). For many authors (e.g., Rogers, 1961), honesty (i.e., authenticity, integrity) is perceived as a fundamental aspect of well-being and a healthy functioning; departures from honesty are seen as reflecting psychopathology. In fact, Wood et al. (2008) found a negative association between authentic living and negative affect. Open-mindedness involves questioning our own thoughts and beliefs, and being able to change our mind in light of evidence. This is, in fact, the core of many psychological treatments (e.g., cognitive behavioral therapy). Open-mindedness reflects a high psychological flexibility, which has been proposed as the very essence of health (Kashdan and Rottenberg, 2010). When focusing on the lowest correlations with negative affect, spirituality/religiousness, modesty, appreciation of beauty and excellence, prudence and fairness occupied the last five positions in the ranking (all correlations were non-significant), which is highly consistent with previous studies and with our assumptions (e.g., Littman-Ovadia and Lavy, 2012; Azañedo et al., 2014).

When considering all components of subjective well-being jointly, the first five strengths most highly correlated with subjective well-being were hope, zest, humor, love, and perseverance, while modesty, spirituality/religiousness, appreciation of beauty and excellence, prudence, and fairness in general showed the lowest correlations with subjective well-being. However, this should not be interpreted automatically as if these strengths were not important for well-being. It is necessary to keep in mind that the indicators of well-being we used in this study focus on the subjective well-being of the individual. So although strengths such as prudence or modesty may not be related to the subjective well-being of the individual directly, as, for example, emotional strengths do, they might be fundamental for a healthy community life, which in turn affects the subjective well-being of the individual. Overall, correlations between strengths and positive affect and life satisfaction, i.e., the positive components of subjective well-being, were slightly larger than with negative affect, a finding that has been already observed and that is consistent with our assumptions (i.e., Littman-Ovadia and Lavy, 2012).

When focusing on the second goal of this study, i.e., the examination of the relationship between strengths and well-being across age groups, we observed that in general these associations seem to slightly increase with age. This is consistent with Isaacowitz et al.'s (2003) results and with our hypotheses. Although the difference is small, this finding suggests, as Isaacowitz et al.'s (2003) suggested, that older adults, who are freed from family and professional constraints, might have more opportunities to apply strengths and, thus, benefit more from them. On the other hand, consistent with Erikson's account of development, it could be possible that, as individuals age, more functions are to be met, and more strengths might be helpful in fulfilling these different functions. These conditions would be reflected in a larger relationship between strengths and well-being. When comparing the size of the correlations between each strength and each component of well-being across the three age groups, a few differences were statistically significant. Specifically, the correlation between creativity and positive affect was significantly larger in the 47–57 years group than in the 27–36 years group. Also, the correlation between forgiveness and positive affect was significantly larger in the 47–57 years group and in the 37–46 years group than in the 27–36 years group. Likewise, the correlation between religiousness and positive affect was significantly different in the 27–36 years group than the correlation in the 37–46 years group and in the 47–57 years group. In fact, the negative significant correlation between positive affect and spirituality/religiousness in the 27–36 years group was an unexpected result that merits our attention. A possible explanation is that spirituality/religiousness is not a source of positive affect for this age group because society, increasingly secular and hedonistic, fails to provide enough opportunities to apply this strength.

When focusing on the relative dominance of some strengths in comparison with others, in the ranking of their relationship with life satisfaction, positive affect and negative affect, in each age group, this ranking was somehow different for the three age groups examined. Overall, for the three age groups, hope, zest and humor were among the first five strengths in the ranking of the relationship with well-being. This is consistent with our assumption that hope is relevant through the life span. However, also consistent with our hypotheses, in the 27–36 years group, which is roughly equivalent to the young adults in Erikson's theory (1982), strengths more related to the promotion of affiliation and commitment with others seemed to be important for well-being. Specifically, strenghts such as kindness, honesty, social intelligence, and teamwork, occupied the first positions in the ranking of the association between strengths and the different components of subjective well-being. They also occupied the first positions when considering the mean absolute value of these three components. Nonetheless, these strengths did not occupy the first positions of this ranking in the 37–46 and 47–57 years groups. Also in line with our expectations, in the 37–46 years group (roughly equivalent to the adults in Erikson's theory), - along with hope, zest and humor - strengths that promote the maintenance of the world (i.e., family, work), such as leadership, love and perseverance, occupied dominant positions in the ranking of the association between strengths and the different components of subjective well-being. This was true not only with respect to the three different components of subjective well-being but also to the mean absolute value of these components. However, these strengths did not occupy the first positions of this ranking in the 27–36 and 47–57 years groups. Finally, again in agreement with our hypotheses, in the 47–57 years group, which could be somehow related to the old adults in Erikson's theory, besides hope, zest and humor, strengths that facilitate integration and a vital involvement with the environment, such as gratitude and love of learning, were among the first five strengths in the ranking of the association between strengths and the different components of subjective well-being, as well as with the mean absolute value of these three components. Nevertheless, these strengths did not occupy the first positions of this ranking in the 27–36 and 37–46 years groups. Older adults naturally begin to look backwards and remember episodes of their life (Butler, 1963). Gratitude might be useful in this process, as it might allow for a positive reinterpretation of the past (Wood et al., 2007) and facilitate integration. Love of learning might be more dominant in the ranking in this stage of life because individuals, more free from family and professional demands have more opportunities to develop their interests and hobbies, which contributes to an active lifestyle (Isaacowitz et al., 2003). Also, the Spearman correlations showed how the rank order of the relationships between strengths and well-being is less similar for widely separated age groups, i.e., for the 27–36 years group and the 47–57 years group. Overall, these results could be suggesting a gradual change in this rank order as individuals age.

Although the sample we used is representative of adults of working age living in Switzerland, one of the limitations of this study is that it does not include young participants below 27 or adults above 57. A sample that includes participants in these age groups would provide a more detailed account of the possible evolution of the association between character strengths and well-being. Also, given the cross-sectional nature of the age comparisons used in the study, it is not possible to conclude that the differences found are due to developmental trajectories. They could also be due to cohort effects. Future longitudinal studies could provide more evidence in relation to the differences in the association between character strengths and well-being over time. Regarding the size of the correlations between strengths and well-being, lower correlations were observed in comparison with other studies using the VIA-IS (e.g., Ruch et al., 2010). A possible explanation for this finding is that the CSRF uses only one item to assess each strength. This is a limitation of the instrument used, which, however, is particularly suited for large-scale longitudinal studies in which large samples compensate for a lower reliability, and economy of instruments is at a premium, as it was the case in this study.

Character is associated with well-being, and some strengths consistently seem to yield higher correlations with well-being than others. Until now, most studies had focused on samples whose representativeness was ill defined. In this study, with a more representative sample, some differences emerged with regard to previous studies, although hope, zest and love consistently yield the highest correlations with life satisfaction, also across different age groups. This finding is consistent with previous research. Future studies will indicate whether these findings are stable across other representative samples in other countries. Moreover, this study suggests the importance of considering age when studying the relationship between strengths and well-being, as some strengths might be particularly important for specific life stages. This relationship may have relevance for further research and practice.

### Conflict of interest statement

The authors declare that the research was conducted in the absence of any commercial or financial relationships that could be construed as a potential conflict of interest.
